# The Associated Study of Crime and Insanity

**Published:** 1897-03

**Authors:** 


### THE ASSOCIATED STUDY OF CRIME AND INSANITY.

Dr. Edwin Goodall, medical superintendent Joint Counties Asylum, Carmarthen, in an article on this subject in the *Lancet*, (No. 3826), calls attention to the very close relations existing between the two classes. The desirability of having prison medical officers experienced in the management of the insane, and vice-versa, is fully and thoroughly discussed, and the danger that, acting apart, these two classes of medical men should leave intact the root of the neurotic tree, whilst they are busily engaged in "lopping off its branches," pointed out. He says:

"Recently, attempts have been made to show that the prison system is productive of insanity, and that a considerable amount of the insanity recorded in prisons is due to the treatment therein received. An unbiased inquiry into the cases so recorded would probably show that in the vast majority the insanity manifested itself shortly after admission; in other words, that in the great majority of the cases, the individual was either actually insane, or a candidate for insanity, on admission to jail."

Prominent among irresponsible persons who arrive in prison, in consequence of the excessive regard paid to the offense and the inadequate estimate of the offender, is the general paralytic in an early stage. In the absence of expert supervision of jails, his malady is likely enough to be overlooked. "Crime is a medical question, and candidates for medical appointments in prisons should be required to show that they have given special attention to lunacy."

England is behind continental Europe in its knowledge of criminals. "Alike abroad and at home the question arises, whether it would not be advantageous to associate with each other the prison and asylum services, in order that crime and insanity may be studied side by side."

